# Impact of Spent Mushroom Substrates on the Fate of Pesticides in Soil, and Their Use for Preventing and/or Controlling Soil and Water Contamination: A Review

**DOI:** 10.3390/toxics4030017

**Published:** 2016-08-17

**Authors:** Jesús M. Marín-Benito, María J. Sánchez-Martín, M. Sonia Rodríguez-Cruz

**Affiliations:** Institute of Natural Resources and Agrobiology of Salamanca (IRNASA-CSIC), 40-52 Cordel de Merinas, 37008 Salamanca, Spain; jesusm.marin@irnasa.csic.es (J.M.M.-B.); mjesus.sanchez@irnasa.csic.es (M.J.S.-M.)

**Keywords:** spent mushroom substrate, pesticide, soil, behavior, field and laboratory experiments

## Abstract

Intensive crop production involves a high consumption of pesticides. This is a cause of major environmental concern because the presence of pesticides in water is becoming increasingly common. Physicochemical methods based on soil modification with organic residues have been developed to enhance the immobilization and/or degradation of pesticides in agricultural soils, which may control both the diffuse and the point pollution of soils and waters. This review summarizes the influence of spent mushroom substrate (SMS) on the environmental fate of pesticides when both are simultaneously applied in agriculture. The processes of adsorption, leaching and dissipation of these compounds in SMS-amended soils were evaluated at laboratory and field scale. Relationships were established between the experimental parameters obtained and the properties of the soils, the SMS, and the pesticides in order to determine the effect that the application of SMS in agricultural soils has on the environmental impact of pesticides. Accordingly, this review highlights the use of SMS as a strategy for the prevention and/or control of soil and water contamination by pesticides to strike a balance between agricultural development and the use of these compounds.

## 1. Introduction

The simultaneous addition of pesticides and organic amendments to soils is a common farming practice in agriculture today. Pesticides increase crop yields and protect them from pests and organic amendments, preserving soil health and quality. On the one hand, this involves a high consumption of pesticides to ensure food supply for the world’s growing population (currently over 7.4 billion people, with this figure expected to rise to 9.7 billion by 2050); on the other, an increase in the organic matter (OM) content of soils is required to improve their fertility and avoid their degradation by agricultural practices [1,2,3].

Agricultural practices include the application of pesticides to eliminate pests and diseases from crops. Nowadays, farmers consider pesticides to be essential compounds for controlling the pests and diseases that threaten our food supply. Oerke and Dehne [1] estimate losses in most crops of between 26% and 40% due to either undesirable pests or the competition between crops and weeds for soil nutrients. Given the importance of chemical crop protection products in agricultural yields, large investments are made annually throughout the world. In 2012, the sales of pesticides reached $47.26 billion [2]. Among a wide variety of pesticides, the highest percentages of application correspond to herbicides (48.5%), followed by fungicides and bactericides (26.6%), and insecticides (18.9%) [3].

Modern pesticides are more powerful and selective than older ones, so ever lower doses may be used. However, the fate of these compounds is a cause of considerable environmental concern because the use of mobile and/or persistent pesticides affects soil and water quality [4,5]. In this sense, the contamination of water by pesticides is increasing in agricultural areas around the world, and in some cases they record higher concentrations than the limit established for drinking water by EU legislation (0.1 µg·L^−1^) [6,7,8,9]. Diffuse (non-point) and point contamination of soil and water by pesticides are frequent in agriculture, as indicated by different authors [10,11,12].

Point source pollution is usually caused by an inappropriate handling of pesticides during their storage and use, or during the equipment cleaning process after application [13,14]. On the other hand, diffuse source contamination is often linked to the use of pesticides in agricultural practices. There are a number of differences between both kinds of contamination sources. Whereas point sources are characterized by high pesticide loads in limited areas, diffuse sources are described by low pesticide concentrations over large areas. Therefore, the reported ways of minimizing or avoiding the risk of contamination by diffuse and point sources are different. Green agricultural practices are applied in the case of diffuse contamination, while other physicochemical systems or strategies are implemented to avoid point contamination [13,15]. However, both strategies are based on the same physicochemical processes, namely, the modification of pesticide behavior in soils after the application and/or use (as components of the biobed biomixtures) of different organic wastes with a high OM content [16,17].

Our modern lifestyle means that high amounts of different organic residues are generated from urban, agricultural, livestock and industrial activities. For instance, more than 1.8 × 10^3^ million tons of waste are generated annually in Europe, which is the same as 3.5 t/person [18]. In recent years, the accumulation of waste is an ongoing concern, not only from an environmental perspective, but also in terms of the risk to human health. Thus, a proper management of these wastes is of vital importance, and giving them an alternative use is a priority in many countries. Different strategies have been reported for valorizing and recycling such materials as organic amendments in soils [18].

Soil amendment with organic residues is a widespread practice in modern agriculture whose main aim is to preserve soil fertility and its present and future agronomic value. This practice seeks to exploit all the nutrients (micro- and macronutrients) that the organic amendment contains, mainly its high OM content, to maintain or raise it in soils with low OM content (<2%). Moreover, these residues improve or maintain the soil´s physical and hydrological properties, since they decrease bulk density, increase water-holding capacity, cation exchange capacity, the aggregation and structural stability of the soil, and its global porosity, together with the modification of pore size and connectivity. In addition, organic residues favor gas and water exchange in the soil, the exploratory capacity of plant root systems, and the development of the soil´s bacterial flora. All this helps to protect the soil against physical processes such us run-off or erosion, and improve the revegetation of degraded soils [19,20]. On the other hand, some authors have also reported that soil amendments may be considered a way of capturing carbon dioxide from the atmosphere [21,22]. Accordingly, the use of organic residues as amendments has important social and agricultural benefits. However, the solid and liquid OM of these organic wastes may modify the physicochemical behavior of pesticides in soils (adsorption-desorption, mobility, degradation, etc.) affecting soil quality and surface and ground waters when these compounds are applied to the soil [23].

Among the organic residues potentially applicable to soil as organic amendments are those from urban (sewage sludge or urban solid wastes), agricultural (crop residues), livestock (manure and slurry) and agro-industrial activities (wine, beer and olive production, and mushroom cultivation). In fact, the modification of the fate of pesticides by some of these residues has already been studied [24,25,26]. In recent years, spent mushroom substrate (SMS) has been addressed in numerous studies on its application to soil, with or without previous composting, due to its high OM and low toxic elements contents, making it an attractive organic amendment [27]. SMS is generated in high amounts in the production process (5 kg of SMS are generated for 1 kg of mushroom produced), with the major producers of mushrooms being China (7,076,842 t), followed by Italy (792,000 t), USA (406,198 t), the Netherlands (323,000 t), Poland (220,000 t), Spain (149,700 t), and France (104,621 t) in 2013 [3]. This is a complex organic residue generated following mushroom cropping. This heterogeneous composite includes the initial compost material used as base in the production of mushrooms, with a greater or lesser degree of development depending on the cultivated mushroom and the subsequent process of alteration or composting it undergoes.

SMS might be considered a potential candidate for soil amendment and/or pesticide control. It is inexpensive and readily available in mushroom-producing countries. Different types of SMS have been tested as organic amendments in soils. Their characteristics vary depending on the nature of the components (normally wheat straw, chicken manure, water, ammonium nitrate, urea, gypsum, calcium carbonate, wood sawdust and plant residues, among others) and of the treatment prior to application. Some of these residues are used fresh immediately after being discarded from mushroom production, whereas others are used after composting under aerobic conditions designed to homogenize and stabilize them. There are several uses for SMS: as a source of lignocellulosic enzymes, animal feed, energy feedstock, dye decolorization, biosorbent of inorganic and organic contaminants, and biodegradation and bioremediation of organopollutants, including pesticides, in soils [27]. The application of SMS to a soil with low OM content is a way of valorizing this residue, and this practice could have positive effects on the soil microbial communities responsible for preserving soil properties [28]. SMS application increases OM content and improves soil fertility and structure, so it may be considered a good strategy for rehabilitating soil quality [29]. Table 1 provides a summary of the main SMSs reported in the literature and their characteristics.

The objective of this review is to summarize the effect of SMS on the behavior of different pesticides (Table 2) [36], and its possible application as a physicochemical strategy for preventing and controlling soil and water contamination by pesticides.

## 2. Application of SMS to Soils and Its Effect on the Behavior of Pesticides

### 2.1. Effect of SMS on Pesticide Adsorption-Desorption

The adsorption-desorption process is considered of great interest among the different processes controlling the dynamics of pesticides in soils because it governs their environmental fate when applied to soils. This process has a direct or indirect influence on the availability of pesticides to be transported to surface or ground waters by run-off or leaching, respectively, transferred to the air by volatilization, taken up by plants, or transformed by soil microorganisms [37]. Accordingly, a high adsorption of this kind of compounds hinders their leaching, run-off, volatilization, and even their biodegradation, while a weak adsorption or a wide desorption of the pesticides favors such processes [38,39,40]. Based on pesticide behavior and on the need to revalorize the huge amount of SMS generated annually, the literature has reported the effect of SMS application on the adsorption and/or desorption of pesticides when using it as a physicochemical strategy to prevent or control soil and water contamination.

The retention process of a wide number of pesticides (22 compounds, Table 2), mainly fungicides, on soils modified by SMS has been reported [15,30,31,32,33,34,35,41,42]. In these studies, different variables, such as the SMS amendment rate, the type of SMS in terms of nature and treatment (composted or fresh), and the SMS-soil incubation time, have been studied to analyze the modification of pesticide adsorption/desorption when they are applied together with the SMS in soils. The pesticides reported belong to different chemical groups, and their water solubility and hydrophobicity varies within a wide range (from 0.2 to 26,000 mg·L^−1^ for water solubility, and from 0.67 to 4.65 for log *K*_ow_) (Table 2), whereby important and broad conclusions about the influence of SMS in pesticide immobilization were obtained when this residue was used as an organic amendment (Table 3).

The potential capacity of SMS as an adsorbent of pesticides was assessed by Marín-Benito et al. [31]. The study reported the adsorption-desorption capacity of the fungicides metalaxyl, benalaxyl, penconazole, tebuconazole, pyrimethanil, cyprodinil, azoxystrobin and iprovalicarb by the SMS generated after the production of three types of mushrooms, *Agaricus bisporus* (total organic carbon (OC) 28.4%, dissolved organic carbon (DOC) 3.83%, polarity inde*x* (PI) 0.592, humic acids/fulvic acids (HA/FA) 1.02), *Pleurotus* spp. (OC 38.3%, DOC 6.27%, PI 0.793, HA/FA 0.34) and *Lentinula edodes* or shiitake (OC 31.2%, DOC 10.8%, PI 0.746, HA/FA 0.49). Three fresh substrates from *Agaricus bisporus*, *Pleurotus* spp., and shiitake production were used without any composting, and the SMS from *Agaricus bisporus* cultivation was also used after composting (OC 26.4%, DOC 1.19%, PI 0.443, HA/FA 2.82). The values of the high adsorption coefficients *K*_f_ and distribution coefficients *K*_d_ were determined for all the fungicides and for each SMS substrate, ranging from 13.0 to 1385 (*K*_f_) and from 9.65 to 698 (*K*_d_). The highest fungicide adsorption capacity in all cases was observed for the composted *Agaricus bisporus* SMS. The characteristics of the organic materials constituting the SMS, such as the higher OC humification degree or the PI together with the *K*_ow_ value of the fungicides, explained almost 80% of the variability in *K*_d_ values normalized to the OC content (*K*_oc_). However, variables such as DOC and OC contents, which often have a major influence on the adsorption of pesticides by amended soils [23], did not do so here. On the other hand, our desorption results show that the four SMSs record a significant efficiency for the adsorption of the highest hydrophobic compounds studied (cyprodinil, penconazole, and tebuconazole) (Table 2), with the remaining percentage of fungicide adsorbed by SMS ranging from 20% (tebuconazole) to 80% (cyprodinil) after four desorption cycles. However, based on the highest Freundlich coefficient of desorption (*K*_fd_), values for the desorption isotherms of all the fungicides from the composted *Agaricus bisporus* SMS, the authors conclude that this SMS could be used to prevent water contamination by pesticides by immobilizing them in the soil.

Different studies have therefore been carried out comparing the changes in the retention degree of pesticides in unamended and SMS-amended soils. For example, Marín-Benito et al. [30] studied the influence on metalaxyl and penconazole adsorption of adding fresh and composted SMS (25 t SMS ha^−1^) (from *Agaricus bisporus* cultivation) to soils. A higher adsorption of both fungicides by the SMS-amended soils was reported. However, whereas the fresh SMS, with a higher OC content, had a higher adsorption capacity of penconazole than the composted SMS, this was not the case for metalaxyl, with no significant differences between fresh and composted SMS. The adsorption of both fungicides was not apparently influenced by the presence of DOC, as indicated by other authors [35,43,44,45].

The potential use of organic amendments as a physicochemical strategy for the immobilization of pesticides in soils depends on their effect on the adsorption-desorption process of these compounds, not only when actually applied, but also over the long term. In this sense, Marín-Benito et al. [32] assessed the evolution of the adsorption-desorption of the fungicides metalaxyl, penconazole, pyrimethanil and iprovalicarb over time in a SMS-amended soil. The soil (OC 0.59%) was amended at two different rates (25 and 125 t SMS ha^−1^) with fresh SMS from shiitake production (OC 31.2%, DOC 10.8%, HA/FA 0.49) and with composted SMS from *Agaricus bisporus* (75%) and *Pleurotus* spp. (25%) production (OC 26.7%, DOC 1.22%, HA/FA 2.43). On the one hand, the results show an increase in the adsorption (*K*_d_) of the four fungicides by the SMS-amended soils, with this increase being higher for the soils amended with (*i*) the composted SMS, and (*ii*) the higher dose of SMS. Independently of the amendment rate, *K*_d_ values increased in parallel to the hydrophobicity of the fungicides. The results reveal a higher adsorption capacity of fungicides by soils with lower OC and DOC content, and with a higher degree of OC humification (HA/FA), according to the major increase in adsorption observed for soil + composted SMS with regard to soil + fresh SMS. On the other hand, a decrease in the adsorption of fungicides was observed when SMS-amended soils were incubated for six and 12 months under laboratory conditions. This behavior was attributed to the decrease in OC content observed in the amended soils over time, and not to the changes in their nature or structure linked to the increase in OC humification determined after 12 months of incubation with regard to the initial values. With regard to the desorption process, SMS addition hindered penconazole and pyrimethanil desorption, whereas it increased for metalaxyl and iprovalicarb. Incubation time had the opposite effect on fungicide desorption. Nevertheless, the total desorption of fungicides was never observed.

The effect of the incubation time of SMS + soils on the adsorption of linuron, diazinon and myclobutanil has also been studied by Rodríguez-Cruz et al. [35]. Three different soils were used in the experiment (OC 0.47%–0.82%), and in this case, the organic residues + soils were incubated outdoors for one month and 12 months. The SMS used was composted from *Agaricus bisporus* (75%) and *Pleurotus* spp. (25%) production. The results reveal that the soil adsorption capacity of pesticides increased after the application of the amendments. The higher adsorption capacity of SMS-soils was observed for myclobutanil, and the adsorption was not related to pesticide hydrophobicity. There was a decrease in the adsorption process for linuron and diazinon, explained by the decrease in OC content with incubation time. The soil adsorption capacity of pesticides was influenced more by their OC content than by OC nature or structure. However, higher adsorption was determined for myclobutanil over time, revealing that the OC structure was more decisive in the adsorption process than the OC content. Accordingly, the effect of the modifications of the OC structure over time on the adsorption of pesticides by SMS may not be widespread because it depends on the chemical structure and properties of these compounds. 

The same composted SMS applied to a sandy loam soil (OC 1.37%) at rates of 50 and 150 t SMS ha^−1^ was used by Herrero-Hernández et al. [33] to assess the changes in the adsorption of fungicides with the incubation time of SMS + soil at field scale. On the one hand, the authors observed that the adsorption of azoxystrobin was higher in the SMS-amended soils, consistent with the increase in OC content after SMS addition. On the other hand, the adsorption of azoxystrobin decreased in the amended soils over time. This decrease was linked to changes in the OC content over time, as reported in the literature for other fungicides in SMS-amended soils incubated under laboratory conditions [32]. Conversely, Herrero-Hernández et al. [34] observed that the addition of composted SMS (from *Agaricus bisporus* (75%) and *Pleurotus* spp. (25%) production) to a sandy loam soil (OC 1.31%) in the field at rates of 40 and 100 t SMS ha^−1^ increased the adsorption of tebuconazole, not only after the addition of the residue, but also over time despite the decrease in the OC content of the amended soils. The decrease in DOC content over time was cited as a possible cause of tebuconazole behavior.

In order to prevent and/or control diffuse and/or point water contamination by pesticides, Álvarez-Martín et al. [15] assayed the use of SMS (from *Agaricus bisporus* cultivation) as a biosorbent, applied at different rates (2%–75% *w*/*w*), of non-polar pesticides (tebuconazole and triadimenol) and polar ones (cymoxanil and pirimicarb) in three soils (OC 0.67%–1%). An increase was observed of up to three times in the adsorption (*K*_d_) of all the pesticides in the amended soils for rates of SMS between 2% and 10%, and up to 20 times for rates from 25% to 75%. The results indicate that SMS may be used as an amendment at rates lower than 10% *w*/*w* to combat the diffuse contamination linked to the common use of pesticides in agriculture, and at rates higher than 25% to minimize point contamination of water by pesticides, together with other known alternatives, such as biobeds.

Furthermore, some authors have also tested the effectiveness of different types of SMS as an adsorbent of pesticides when used as biomixtures. For instance, Karanasios et al. [41] observed an increase in the adsorption of the pesticides metalaxyl-M, terbuthylazine, metribuzin and indoxacarb in SMS (composted *Agaricus bisporus*) biobeds compared to soil (*K*_f_ values increased by 1.3 to 7.7 times). In addition, the results showed that the percentages of desorption were below 30% of the amount adsorbed for all the pesticides, being in some cases very low (0.36% for indoxacarb) or even zero (terbuthylazine). Karas et al. [42] assessed the adsorption and dissipation of the pesticides thiabendazole, imazalil, ortho-phenylphenol, and diphenylamine in biobeds packed with organic biomixtures including SMS (*Pleurotus ostreatus*) as substrate, and also concluded that this method could be used successfully for the treatment of wastewaters, as compared to soil the SMS biomixtures had a higher adsorption and dissipation capacity for all the pesticides tested. Table 3 includes a summary of the adsorption and/or desorption of pesticides by SMS alone and by SMS-amended soils.

### 2.2. Effect of SMS on Pesticide Mobility

The mobility of a pesticide in the soil may involve leaching or vertical movement in the soil profile or by run-off when water exceeds its infiltration capacity. The leaching process is considered to be the main cause of groundwater contamination by pesticides, while surface waters may become contaminated when these compounds are transported by run-off. Both processes depend mainly on the physicochemical properties of soil and pesticides, which determine their adsorption by soil components [37]. Pesticides with an intermediate adsorption rate are more likely to suffer losses by run-off, since the weakly adsorbed compounds are rapidly leached through the soil from the surface. In general, the greater adsorption of pesticides means they are less available for leaching. Thus, lower leaching would be expected in soils with a high OM content due to greater adsorption [46]. Soil OM content could be increase by adding organic amendments to soils. Previous works have reported the adsorption of pesticides by the OM of amended soils, and a reduction in their transport through the soil profile [47,48]. However, in other works, the increased OM in amended soils favored the transport of pesticides [44,49]. A reduction in pesticide adsorption due to the higher DOC content of amended soils could favor pesticide mobility due to: (i) the adsorption of pesticides by DOC; (ii) the competition for adsorption sites; and/or (iii) interferences in the adsorption of pesticides by soil components [23,49]. Furthermore, the changes in soil porosity due to the amendment and the higher OC content could explain the changes in pesticide mobility in amended soils [50].

The application to agricultural soils of SMS containing high solid OC and DOC could modify pesticide behavior and alter their retention by soils or their transport to surface and ground waters. Both processes could be affected when SMSs are used as a soil amendment, depending on their nature and the doses applied. Studies on pesticide mobility in SMS-amended soils have been reported for compounds with different properties (Table 2). They involve laboratory assays using undisturbed soil cores taken directly in the field to maintain soil macrostructure within the soil profile [51], or packed soil columns [52,53,54]. Studies of pesticide mobility in both types of columns have been performed to ascertain the effect of SMS on the leaching of pesticides after a continuous flow of water or aqueous solution of CaCl_2_ 0.01 M (saturated flow) or a discontinuous flow (saturated-non saturated flow) has been applied to the column. Table 4 includes a summary of the results.

#### 2.2.1. Pesticide Mobility in Undisturbed Soil + SMS Cores

The influence of SMS from *Agaricus bisporus* cultivation on the mobility of metalaxyl and penconazole in undisturbed cores taken from vineyard soils unamended and amended at 25 t·ha^−1^ with fresh or composted SMS (F-SMS and C-SMS) with different OC and DOC content (Table 1) has been studied [51]. Experiments were performed under non-saturated flow conditions (Table 4). For metalaxyl, the maximum peak concentration of breakthrough curves (BTCs) decreased in both SMS-amended soils. In the amended soils, maximum peaks were reached at the same or higher pore volume (PV) than in unamended soils. The increased adsorption of metalaxyl by SMS-amended soils, as indicated by the adsorption constants *K*_f_ obtained by Marín-Benito et al. [30], was consistent with the leaching results. The higher DOC content in F-SMS was responsible for the higher leaching of metalaxyl in F-SMS-amended soil, due to interactions in the solution between DOC and the hydrophilic fungicide. The fungicide was retained more in the C-SMS-amended soil, and higher total residual amounts were found in the first soil core layer (0–8 cm). The total balance of metalaxyl ranged between 27.2% and 89.0%, indicating that other processes, such as degradation, mineralization and the formation of non-extractable residues, could occur throughout incubation. These results indicate that the leaching of metalaxyl was mainly controlled by adsorption and biodegradation, due to the application of SMS. The application of SMS might prevent the contamination of groundwater by this fungicide, due to its higher adsorption and lower leaching as observed for other amended soils [43,44,45].

On the other hand, the fungicide penconazole did not leach after the experiment, and it was immobilized in the soils due to its high adsorption coefficients [30,55,56]. The amounts retained in the first 8 cm of the soil core accounted for >60% of the fungicide applied. The retention of penconazole was higher in SMS-amended soils, and it was not degraded during the experiment. These results indicated that penconazole could reach surface water when soil particles are mobilized through run-off [51].

#### 2.2.2. Pesticide Mobility in Packed Soil + SMS Columns

Packed columns of a sandy loam soil (OC 0.47%) were used to study the effect of soil + SMS incubation time for one month or 12 months on the leaching of three pesticides with different characteristics: linuron, diazinon and myclobutanil (Table 2). A comparison with other amendments (sewage sludge and grape marc) was also reported by Marín-Benito et al. [52]. SMS was applied at a rate of 5% *w*/*w* or 25 t·ha^−1^, and the OC and DOC contents were 2.18% and 0.036%, and 1.95% and 0.007% for non-incubated (one month) and incubated (12 months) SMS-amended soils, respectively. The DOC and humification degree were evaluated over time. The SMS-amended soil recorded higher immobilization, and pesticide leaching decreased due to the higher OC content. The results indicate that the adsorption of linuron and myclobutanil by SMS-amended soils was higher than by other amended soils, but the effect was relatively small for the insecticide diazinon. The effect of incubation time was not observed on the BTCs of pesticides, and the leaching of the pesticides studied in SMS-amended soils was still reduced by the amendment after incubation. However, the retention of linuron and diazinon over time depends more on soil OC content than on humification degree [35].

Packed columns of a sandy clay loam soil with low OC content (OC 0.67%) were also used to study the SMS effect applied at a low rate (5% *w*/*w*), simulating its application to soil as an organic amendment, and at a high rate (50% *w*/*w*), simulating its application to soil as a barrier on the mobility of tebuconazole and cymoxanil, under different flow conditions (saturated or saturated-non-saturated) [53,54]. The OC content was 1.73% for S + SMS5, and 16.3% for S + SMS50. The results indicate that the leached amounts of tebuconazole in unamended soil decreased by up to two or three times (S + SMS5 and S + SMS50) when a water volume corresponding to 12 PV was applied as saturated flow. These amounts also decreased in S + SMS5 when a saturated-non-saturated flow was applied, but it did not significantly modify the fungicide leaching in S + SMS50. The amounts leached agree with the amounts retained, and > 50% of fungicide was found in the first segment in the column under all the conditions studied in S + SMS50. Cymoxanil leached faster than tebuconazole from unamended and SMS-amended soils. The total leaching of cymoxanil after applying 12 PV to the column was ≈100% for saturated flow, with no retention of the fungicide in the column, and it was lower when saturated-non-saturated flow was applied to the column (91.9% (S), 72.5% (S + SMS 5) and 36.8% (S + SMS 50)). Cymoxanil was not retained by the unamended and SMS-amended soil when a saturated flow was applied, but retained amounts of <50% were found in the first segment of S + SMS5 and S + SMS50 columns under saturated-non-saturated conditions. Both fungicides were retained mainly in the amended soil according to the increases in the adsorption constants of tebuconazole and cymoxanil by SMS-amended soil [15]. SMS decreased tebuconazole leaching by increasing the adsorbed amount in a non-extractable form, and decreased cymoxanil leaching by increasing its adsorption and decreasing its mineralization. 

The SMS from *Pleurotus ostreatus* cultivation, either alone or mixed with straw and soil, also showed great potential when used as a column filling for retaining and dissipating fungicides such as ortho-phenylphenol and imazalil. Fungicide leaching decreased to <1% and <0.5% of their initial amounts, respectively, from the wastewater of citrus fruit-packaging plants with high fungicide loads [55]. The results evidence the depuration capacity of biobeds with SMS-rich substrates receiving polluted effluents.

#### 2.2.3. Pesticide Mobility in SMS-Amended Soils: Field Experiments

Herrero-Hernández et al. [33,34] have studied the mobility of tebuconazole and azoxystrobin in a sandy loam soil (OC 1.31%) in vineyard field plots amended with SMS at different rates between 40 and 150 t·ha^−1^. The fungicides tebuconazole and azoxystrobin were applied at two doses (0.25 and 1.25 kg·ha^−1^), and soil cores were collected at a depth of up to 50 cm over one year. The analysis of fungicide residues at the different soil depths (0–50 cm) indicated that both fungicides were transported to deeper soil layers, although residue concentrations were much higher in the first layer, and they were also higher in the soil plots treated with the highest doses of fungicides. The leaching of fungicides was higher in the SMS-amended soils than in the unamended one. The soil amended with SMS at ≥100 t·ha^−1^ and treated with a high dose of fungicides underwent the highest fungicide immobilization in the first soil layer (up to 10 cm). The highest OC content of SMS-amended soils explained the higher persistence of both fungicides throughout the soil profile. Accordingly, a significant relationship was found between fungicide amounts and the OC content of soil profiles. In conclusion, SMS was initially responsible for fungicide immobilization on the soil surface, although this was inverted over time, as fungicide leaching increased in SMS-amended soils. Although fungicide amounts throughout the soil profile decreased over time, they were still detected at 50 cm after one year.

### 2.3. Effect of SMS on Pesticide Degradation and Dissipation

The application of organic amendments to soil affects pesticide dissipation. The activity of soil microbial communities can be modified by the addition of the OM and nutrients contained in the amendment. In this sense, the stimulation of soil microbial activity by the amendment may increase pesticide degradation [16,41]. On the other hand, the presence of an organic amendment in the soil favors the adsorption of pesticides, which would decrease their bioavailability for degradation by soil microorganisms [33,37]. The results reported in experiments carried out under field and laboratory conditions (Table 5) indicate that SMS had an effect on the dissipation and bioavailability of pesticides with different characteristics in SMS-amended soils. These studies were carried out to investigate the fate of pesticides (degradation and persistence) in SMS-amended soils. Furthermore, these works reported the ability of SMS to increase or decrease the dissipation rate of pesticides with different characteristics in soils (Table 2).

The effect that SMS applied to soil has on pesticide dissipation and degradation needs to be assessed in order to prevent possible risks of water contamination as a consequence of intensive pesticide application in agriculture. Field studies therefore allow both evaluating pesticide dissipation in SMS-amended soils under realistic conditions and specifying effective amounts of SMS and pesticide. In addition, the results from laboratory studies on pesticide dissipation in SMS-amended soils are needed to explain pesticide fate under controlled conditions.

In general, different factors were taken into account to evaluate the effect of SMS application on pesticide dissipation, such as SMSs with different nature and origin, and the SMS rates or incubation time of the SMS-amended soil. The use of SMS as a physicochemical method to prevent soil and water contamination was also evaluated. On the other hand, the use of SMS as an organic amendment in agricultural soils could not only affect pesticide dissipation, but also the composition and function of the microbial community. In this sense, different studies have used sundry approached to evaluate the impact that SMS and pesticides have on soil microbial activities, such as dehydrogenase activity (DHA), and on soil microbial community structure (Table 5).

#### 2.3.1. Pesticide Dissipation and Mass Balance in SMS-Amended Soils: Field and Laboratory Studies

Herrero-Hernández et al. [33,34] conducted a field and laboratory study over the course of a year to evaluate the dissipation of two fungicides, tebuconazole and azoxystrobin, in an agricultural soil amended with SMS at two rates (Table 5). The study was carried out in experimental plots, and the results show that the dissipation kinetics of tebuconazole fitted a biphasic pattern in the field scenario. Half-life (DT_50_) values were lower in both SMS-amended soils (8.2–10.9 days), and they increased in an unamended soil (11.6–12.4 days) treated with a low dose of fungicide (0.25 kg·ha^−1^) and a high one (1.25 kg·ha^−1^). Although tebuconazole was available for biodegradation in surface soils, only chemical or photochemical degradation was possible over time. The DOC from the SMS amendment had an influence on the persistence and degradation of tebuconazole in amended soils over time [34]. Similarly to tebuconazole, azoxystrobin dissipation kinetics was fitted to a biphasic kinetic model under field and laboratory conditions, and the dissipation rate was lower in unamended soil than in amended ones. The first dissipation phase was shorter under field conditions, and the DT_50_ values were higher under laboratory conditions (89.2 days in unamended soil, and 148 days in SMS amended soil). These results could be explained by the absence of other processes controlling the fate of azoxystrobin under laboratory conditions, which may occur under field conditions (photodegradation, mobility, etc.). The adsorption and leaching of azoxystrobin through the soil profile controlled the dissipation process and conditioned the persistence of this fungicide in SMS-amended soils [33].

Marín-Benito et al. [57] carried out a laboratory study to assess the influence that the nature and treatment of two SMS (composted C-SMS and fresh F-SMS) applied to a vineyard soil had on the dissipation of iprovalicarb, metalaxyl, penconazole and pyrimethanil, and the determination of the mass balance of metalaxyl and penconazole. The dissipation kinetics followed single first order or biphasic kinetic models. The dissipation rates were lower in C-SMS-amended soils than in an unamended soil, and the DT_50_ values were more than a thousand times higher for penconazole. The DT_50_ values of metalaxyl and pyrimethanil in F-SMS soils were similar to those in unamended soils, although they were higher for iprovalicarb and penconazole. These results are related to the adsorption of these fungicides by unamended and SMS-amended soils indicated previously [32]. The immobilization of these compounds by SMS decreased the dissipation rates due to a reduction in their bioavailability. Furthermore, the dissipation of metalaxyl and pyrimethanil in F-SMS-amended soils was related to the soil’s DOC content and DHA. The results from the mass balance study indicate that dissipation mechanisms such as mineralization, residue extraction and the formation of bound residues depended on the type of SMS applied.

A study was also conducted by Marín-Benito et al. [58] to study the influence that the incubation time (one month and 12 months) of the SMS-amended soil had on the dissipation kinetics and formation of metabolites of linuron, diazinon and myclobutanil, as well as determining the mass balance for linuron and diazinon. The dissipation rate increased and DT_50_ values decreased in the SMS-amended soils for all three pesticides, with the highest DT_50_ values being found in SMS-amended soils, when compared with soils amended with sewage sludge or grape marc, due to the higher HA/FA ratio. The dissipation rates of linuron increased in unamended and amended soils incubated for 12 months, but for diazinon and myclobutanil DT_50_ values increased in all the incubated soils. Pesticide dissipation was related to the adsorption of these pesticides by soils reported in a previous work [35]. Mineralization and extraction were higher for diazinon than for linuron. The extractable residues decreased, while the formation of bound residues increased over time for both pesticides. The effect of soil aging on dissipation was consistent with the changes in the adsorption of pesticides by soils after incubation, as described previously [35].

Álvarez-Martín et al. [59] conducted a laboratory study to evaluate the influence of two SMS rates (5% and 50% *w*/*w*) on the dissipation and bioavailability of cymoxanil and tebuconazole in a vineyard soil. Both SMS rates were selected to prevent the diffuse or point pollution of soil (Table 5), as indicated previously [15]. The dissipation rate was higher for cymoxanil than for tebuconazole in unamended and amended soils, and was lower in the unamended soil than in SMS-amended soils. There were no significant differences in the DT_50_ values of both fungicides in the soils amended with SMS at both rates. The mineralization of both fungicides was lower in the SMS-amended soils due to the higher immobilization of cymoxanil and tebuconazole, as reported previously [15]. The formation of bound residues in SMS-amended soils explained the apparent increase in the dissipation rate of both fungicides. The bioavailability of bound residues of tebuconazole in SMS-amended soils was lower than in the unamended soil. The formation of bound residues of cymoxanil in SMS-amended soils was reversible, with mineralization of part of these residues over time.

As already mentioned, both SMS and pesticides affect soil microbial communities, which may have different effects on soil quality. Therefore, Álvarez-Martín et al. [60] studied the modifications of soil microbial communities in an agricultural soil amended with SMS at rates of 2% and 5% *w*/*w*, and treated with the pesticides azoxystrobin or pirimicarb applied at 2 and 25 mg·kg^−1^ rates (Table 5). The dissipation rate of azoxystrobin was lower than that of pirimicarb after 90 days in both unamended and amended soils. The DT_50_ values of azoxystrobin, applied at the low rate, and pirimicarb decreased, while DT_50_ values of azoxystrobin applied at the high rate increased when SMS was added to the soil. Soil DHA and respiration showed that SMS rate, pesticide dose, and incubation time all affected the microbial community’s metabolism. The results obtained from the next generation sequencing indicate that SMS rate and incubation time have significant effects on soil microbial communities, although no impact of pesticides was observed.

#### 2.3.2. Pesticide Dissipation in Biobeds or Biomixtures including SMS as a Component

A series of works [16,41,42,61] have shown that the application of SMS as a component of a biomixture or biobed has an impact on the dissipation of different pesticides (Table 5). Karas et al. [42] have recently assessed the dissipation of thiabendazole, imazalil, ortho-phenylphenol, diphenylamine, and ethoxyquin in various organic substrates composed of soil, straw and SMS (*Pleurotus ostreatus*) in different ratios. The dissipation of thiabendazole and imazalil (DT_50_ = 28 days) was accelerated in SMS/straw/soil (50:25:25) biomixtures, showing that a SMS-rich biobed is an efficient system for treating effluents from the fruit-packaging industry.

Three biomixtures including different SMSs (*Pleurotus eryngii, Flammulina velutipes*, and *Lentinula edodes*) were used by Gao et al. [61] to study the dissipation of chlorothalonil and imidacloprid. Pesticide dissipation and microbial activity were comparable in all the biomixtures, but significant differences were found between biomixtures and the soil control. The microbial activities (phenoloxidase, respiration, and fluorescein diacetate) observed in SMS-biomixtures were higher than in the soil alone, due to more readily available carbon and active mycelium and spores from the cultured mushroom. These authors concluded that SMS was a suitable substitute for peat in the original biobed design.

Karanasios et al. [16] tested the potential use of SMS applied at different rates (5%, 15% and 50% *v*/*v*) as a substitute for straw in biobeds or biomixtures in degradation studies of metribuzin, buprofezin, azoxystrobin, iprodione, dimethoate, indoxacarb, and terbuthylazine applied at 10% of the standard dose level and at the standard dose. All the pesticides degraded faster (lower DT_50_ values) in the biomixture containing 50% SMS. The higher pesticide degradation rate in the SMS-biomixture was proportional to the amount of SMS. The nutrient source from the SMS and the catabolic capacity of enzymatic systems produced by the mushroom could contribute to fast pesticide biodegradation.

In another study, Karanasios et al. [41] assessed the use of SMS produced in southern Europe as a potential alternative to peat in the traditional biomixture used in a biobed. A biomixture of SMS, soil and straw (1:1:2) was used to evaluate the degradation of a mixture of pesticides (dimethoate, indoxacarb, buprofezin, terbuthylazine, metribuzin, metalaxyl-M, iprodione, and azoxystrobin) at two dosage rates. The SMS-biomixture degraded the pesticides applied at high and low doses at faster rates than those observed in soil. The DT_50_ values ranged from 1.2 to 9 times lower, and from 1.7 to 9.5 times lower, respectively, than those observed in soil when the pesticides were applied at high and low doses.

#### 2.3.3. Biodegradation and Bioremediation of Polluted Soils by SMS

Several works have evaluated the use of SMS as a biological source that contains white-rot fungi, nutrients and enzymes for biodegrading and mineralizing different pesticides, such as heptachlor and its metabolite heptachlor epoxide [62], DDT [63], tricyclazole [64], benzene [71], and polycyclic organic hydrocarbons (PAH) [65,66,67,71,72,73,74,75]. Different studies have addressed the application of SMS to bioremediate contaminated soils by organic compounds including pesticides [62,64,68,69] or PAH [65] (Table 5).

Purnomo et al. [63] investigated the bioremediation of DDT-contaminated soil using SMS from *Pleurotus ostreatus* cultivation. The SMS degraded DDT in the soil with an efficiency of 50% after 28 days, and 5.1% of the DDT was mineralized by SMS in contaminated soil after 56 days’ incubation. A similar study was carried out by the same authors to investigate the ability of SMS from *Pleurotus ostreatus* to degrade heptachlor and its metabolite heptachlor epoxide in an artificially contaminated soil. The SMS degraded 91% of heptachlor and 26% of heptachlor epoxide after 28 days’ incubation [62]. The degradation of tricyclazole in both soil and an SMS-soil mixture was investigated by Liu et al. [64]. The results showed a poor bioavailability of the fungicide in soil, and only 10% of the tricyclazole added was degraded after 56 days. However, a tricyclazole degraded more in the SMS-soil mixture, where 32.8% of the fungicide was dissipated after 56 days. The addition of SMS to soil could accelerate the degradation of this fungicide.

Other works have reported the use of SMS to biodegrade PAH in polluted soils. Reid et al. [65] investigated the biodegradation of phenanthrene in a subsoil amended with SMS from *Agaricus bisporus* cropping. They reported that total phenanthrene loss was significantly greater in SMS-soil mixtures (36.7%–42.7%) than in soil (27.5%) after 111 days of incubation. In two similar works, García-Delgado et al. [66,67] studied the effect that the SMS from *Agaricus bisporus* had on the bioremediation of a soil multi-polluted with PAH and Pb beside a shooting range, and a historically polluted soil with PAH from a creosote wood treatment plant. The best remediation treatment involved the use of SMS sterilization and further *Agaricus bisporus* inoculation to remove PAH, mainly benzo(a)pyrene, and decontaminate the multi-polluted soil [66], and the addition of sterilized SMS to the historically polluted soil to stimulate bacterial population and ensure high degradation of 3-ring PAH [67]. In these studies, SMS was suitable for the bioremediation of soil polluted by PAH and metals. 

The potential of SMS to increase soil microbial activity (DHA) and assist in the remediation of chlorpyrifos-contaminated soil was evaluated by Kadian et al. [69]. SMS enhanced the DHA of contaminated soil and the dissipation of chorpyrifos in the SMS-amended soil was 24% higher than in the unamended soil. In a previous work, Kadian et al. [68] found that the application of SMS to soil also enhanced the dissipation of atrazine, and 29.17% of herbicide was dissipated in the SMS-amended soil, which was twice the percentage of atrazine dissipation in the unamended soil (15.28%) after three weeks of incubation. Qin et al. [70] determined the degradation of two fumigants, 1,3-dichloropropene and chloropicrin, in an organic amended soil. Two organic amendments containing SMS in their composition were applied to a sandy loam soil at a rate of 5% (*w*/*w*, dry weight basis). The application of the SMS-amendment to the soil increased the fumigant degradation rate by up to 1.4 times for 1,3-dichloropropene, and by 5.4 times for chloropicrin, decreasing the DT_50_ values of both fumigants in the soil.

## 3. Conclusions

SMS could be used as an organic soil amendment with a dual purpose: (1) enhance the sustainable recycling of this residue, increasing soil quality, and (2) as a method to control the behavior of pesticides when applied jointly with the SMS in soils. The results reported in the literature indicate that different doses of SMS can be applied to soil in order to develop a physicochemical strategy to prevent or control soil and water contamination. This strategy is based on (1) the SMS’s capacity to control pesticide adsorption and/or desorption by soils, and (2) the influence of these processes on the leaching and dissipation/biodegradation of these compounds. On the one hand, the application of a low SMS rate as an organic amendment to soils could help to prevent diffuse water pollution caused by the frequent use of pesticides in agriculture when a composted residue with a high OC content and low DOC content was applied. The characteristics of pesticides should also be taken into account, since the adsorption of hydrophobic compounds by SMS could lead to the formation of bound residues. Soil particles containing these residues may be mobilized into the aquatic ecosystem and contaminate surface water through run-off, or groundwater through leaching. The widely reported statistical relationship between the adsorption and OC content of SMS and *K*_ow_ of pesticides should be evaluated before adding SMS to the soil. On the other hand, the application of high SMS rates as a component of biobeds or as a soil/SMS mixture could prevent point water pollution when the SMS has a lower composting degree and a higher DOC content in order to increase pesticide bioavailability to microorganisms, and enhance its degradation and/or mineralization. Nowadays, the use of SMS as a biological source for the bioremediation of soils polluted with pesticides or other organic compounds such as PAH is a research topic of considerable interest. However, research on the use of SMS as an agricultural amendment or its application in soil bioremediation needs to be broadened through, for example, the study of SMS effects on the behavior of other emergent contaminants.

## Figures and Tables

**Table 1:** Characteristics of main SMS used.

SMS/Parameter ^1^	pH	TOC%	C%	N%	PI	C/N	DOC%	HA/FA	Reference
Composted *Agaricus bisporus*	6.74–7.4	25.9–27.4	27.7	1.95–2.49	0.443	10.4–14.2	1.01–1.19	2.82	Álvarez-Martín et al. (2016) [15]
									Marín-Benito et al. (2009) [30]
									Marín-Benito et al. (2012) [31]
Fresh *Agaricus bisporus*	6.7–6.97	24.5–28.8	29.4	2.36–2.52	0.592	11.3–14.2	1.91–3.83	1.02	Marín-Benito et al. (2009) [30]
									Marín-Benito et al. (2012) [31]
Fresh *Pleurotus* spp.	5.7	38.3	38.3	0.73	0.793	52.4	6.27	0.34	Marín-Benito et al. (2012) [31]
Fresh *Lentinula edodes* or shiitake	4.5	31.2	31.2	1.75	0.746	17.9	10.8	0.49	Marín-Benito et al. (2012) [31,32]
Composted *Agaricus bisporus: Pleurotus* spp. (3:1)	7.1–7.5	26.7–27.1	28.0	2.20–2.24	0.587	12.1–12.5	1.22	2.43	Herrero-Hernández et al. (2015) [33]
									Herrero-Hernández et al. (2011) [34]
									Marín-Benito et al. (2012) [32]
									Rodríguez-Cruz et al. (2012) [35]

^1^ SMC, spent mushroom substrate. TOC, total organic carbon; PI, polarity index; DOC, dissolved organic carbon; HA/FA, humic acids/fulvic acids.

**Table 2:** Characteristics of pesticides studied. Data taken from PPDB [36].

Compound	Group	Type	Log *K_ow_* ^1^	Polar/Non-Polar ^2^	Water Solubility (mg·L^−1^)	*K_foc_* ^3^ (mL·g^−1^)	DT_50_^4^ (days)
Azoxystrobin	strobilurin	fungicide	2.5	polar	6.7	423	78
Benalaxyl	acetylalaninate	fungicide	3.54	non-polar	28.6	4998	49
Chlorothalonil	chloronitrile	fungicide	2.94	polar	0.81	3032	22
Cymoxanil	cyanoacetamide oxime	fungicide	0.67	polar	780	43.6	0.7
Cyprodinil	phenyl pyrimidinamine	fungicide	4.0	non-polar	13	2277	37
Diphenylamine	amine	fungicide/insecticide	3.82	non-polar	25.8	4104	-
Ethoxyquin	quinoline	fungicide	3.39	non-polar	60.0	3208	-
Imazalil	imidazole	fungicide	2.56	polar	184	4753	76.3
Iprodione	dicarboximide	fungicide	3.0	non-polar	6.8	3927	36.2
Iprovalicarb	carbamate	fungicide	3.2	non-polar	17.8	106	15.5
Metalaxyl	acetylalaninate	fungicide	1.75	polar	8400	162.3	36
Metalaxyl-M	acetylalaninate	fungicide	1.71	polar	26,000	78.9	6.5
Myclobutanil	triazole	fungicide	2.89	polar	132	517	560
Ortho-phenylphenol	phenol	fungicide	3.18	non-polar	560	347	4
Penconazole	triazole	fungicide	3.72	non-polar	73	2205	117
Pyrimethanil	phenyl pyrimidinamine	fungicide	2.84	polar	121	301	55
Tebuconazole	triazole	fungicide	3.7	non-polar	36	769	63
Thiabendazole	benzimidazole	fungicide	2.39	polar	30	2091	500
Triadimenol	triazole	fungicide	3.18	non-polar	72	273	250
Tricyclazole	triazolobenzothiazole	fungicide	1.4	polar	596	144	450
Atrazine	triazine	herbicide	2.7	polar	35	174	75
Metribuzin	triazinone	herbicide	1.65	polar	1165	37.92	11.5
Terbuthylazine	triazine	herbicide	3.4	non-polar	6.6	231	75.1
Buprofezin	unclassified	insecticide/acaricide	4.93	non-polar	0.46	5334	50
Chloropicrin	unclassified	insecticide/nematicide	2.5	polar	10,000	60.5	3.0
Chlorpyrifos	organophosphate	insecticide	4.7	non-polar	1.05	8151	50
DDT (1,1,1-trichloro-2,2-bis(4-chlorophenyl)ethane)	organochlorine	insecticide	6.91	non-polar	0.006	151,000	6200
Diazinon	organophosphate	insecticide	3.69	non-polar	60	643	9.1
Dimethoate	organophosphate	insecticide/acaricide	0.704	polar	39,800	28.3	2.6
Imidacloprid	neonicotinoid	insecticide	0.57	polar	610	225	191
Indoxacarb	oxadiazine	insecticide	4.65	non-polar	0.2	6450	17
Heptachlor	organochlorine	insecticide	5.44	non-polar	0.056	24,000	285
Heptachlor epoxide	unclassified	metabolite	4.98	non-polar	0.2	22,485	-
Pirimicarb	carbamate	insecticide	1.7	polar	3100	388	86
1,3-Dichloropropene	halogenated hydrocarbon	nematicide/bactericide	1.82	polar	2485	33.7	9.3

^1^ log K*_ow_*, octanol/water partition coefficient; ^2^ non-polar when log *K*_ow_ > 3.0; ^3^ *K_foc_*, Freundlich adsorption coefficient normalized for soil organic carbon content; ^4^ DT_50_, aerobic soil degradation half-life.

**Table 3:** Influence of SMS on the adsorption-desorption of pesticides by soils.

Pesticide	Soil	SMS Type/Dose	Results ^1^	Reference
Azoxystrobin Metalaxyl Penconazole Pyrimethanil Iprovalicarb Benalaxyl Tebuconazole Cyprodinil	-	Fresh *Agaricus bisporus* (F-Ag) (OC 26.4%, DOC 1.19%) Composted *Agaricus bisporus* (C-Ag) (OC 28.4%, DOC 3.83%) *Fresh Pleurotus* spp.(F-Pl) (OC 38.3%, DOC 6.27%) Fresh *Lentinula edodes* (F-Sh) (OC 31.2%, DOC 10.8%)	Adsorption: *K*_f_ (13.0–1385) and *K*_d_ (9.65–698). *K*_oc_ influent factors: HA/FA and PI of SMS and *K*_ow_ of fungicides. Desorption: C-Ag was the most effective for retention all the fungicides (*K*_fd_ 2.66–1885) F-Ag, F-Pl and F-Sh only for the most hydrophobic.	Marín-Benito et al. (2012) [31]
Metalaxyl Penconazole	Sandy clay loam (OC 0.60%, pH 7.8) Sandy clay loam (OC 1.01%, pH 7.7) Sandy clay loam (OC 1.47%, pH 7.8)	Fresh *Agaricus bisporus* (F-SMS) (OC 28.8%, DOC 3.83%) Composted *Agaricus bisporus* (C-SMS) (OC 27.4% and DOC 1.19%) Dose: 25 t·ha^−1^	*K_f_* adsorption values higher in amended soils (>2.3 times penconazole, >1.3 times metalaxyl). Increased adsorption by F-SMS (penconazole) SMS was not relevant for metalaxyl adsorption.	Marín-Benito et al. (2009) [30]
Metalaxyl Penconazole Pyrimethanil Iprovalicarb	Sandy clay loam (OC 0.59%, pH 7.8)	Composted *Agaricus bisporus: Pleurotus* spp. (3:1) (OC 26.7%, DOC 1.22%) Fresh *Lentinula edodes* or Shiitake (OC 31.2%, DOC 10.8%) Dose: 25 and 125 t·ha^−1^	Adsorption: increased in the amended soils (with higher SMS dose and composted SMS associated to higher degree of OC humification) and decreased in the amended soils (with the incubation time by decreasing the OC content over time). Desorption: increased in the amended soils (metalaxyl and iprovalicarb) and decreased (penconazole and pyrimethanil). Opposite effect with the incubation time.	Marín-Benito et al. (2012) [32]
Linuron Diazinon Myclobutanil	Sandy clay loam (OC 0.69%, pH 7.4) Sandy loam (OC 0.47%, pH 7.9) Sandy clay loam (OC 0.82%, pH 6.5)	Composted *Agaricus bisporus: Pleurotus* spp. (3:1) (OC 26.7%, DOC 1.22%) Dose: 25 t·ha^−1^	*K*_f_ (*K*_d_) adsorption values increased 1.21–1.76 times (1.28−1.52 times) in amended soils. Decreased with the incubation time (linuron and diazinon) or increased (myclobutanil) by changes in the OC content or on the OC structure.	Rodríguez-Cruz et al. (2012) [35]
Azoxystrobin	Sandy loam (OC 1.37%, pH 7.7)	*Agaricus bisporus: Pleurotus* spp. (3:1) (OC 27.1%, DOC 1.22%) Dose: 50 and 150 t·ha^−1^	*K*_f_ adsorption values increased in amended soils (S + SMS50 0.9−4.2 times, S + SMS150 4.7–34.3 times) and decreased in the amended soils with the incubation time (S + SMS50 4.9 times, S + SMS150 7.4 times after 378 days) by decreasing the OC content.	Herrero-Hernández et al. (2015) [33]
Tebuconazole	Sandy loam (OC 1.31%, pH 7.7)	*Agaricus bisporus: Pleurotus* spp. (3:1) (OC 27.1%, DOC 1.22%) Dose: 40 and 100 t·ha^−1^	*K*_f_ adsorption values increased in amended soils (S + SMS40 2.65−7.03 times, S + SMS100 5.8−9.5 times) and over time (S + SMS402 65 times, S + SMS100 1.64 times after 355 days) associated to the decrease in the DOC content.	Herrero-Hernández et al. (2011) [34]
Tebuconazole Triadimenol Cymoxanil Pirimicarb	Sandy loam (OC 0.89%, pH 7.49) Sandy clay loam (OC 0.67%, pH 7.52) Clay loam (OC 1.0%, pH 7.84)	Fresh *Agaricus bisporus* (OC 24.5%, DOC 1.91%) Dose: 2, 5, 10, 25, 50 and 75% (*w*/*w*)	*K*_f_ adsorption values increased in amended soils for all pesticides with the SMS dose applied (1.41−19.2 times tebuconazole, 0.9−21 times triadimenol, 1.69−28.5 times cymoxanil and 1.51−44.5 times pirimicarb)	Álvarez-Martín et al. (2016) [15]
Metalaxyl-M Terbuthylazine Metribuzin Indoxacarb	Sandy clay loam (OC 1.8%, pH 6.57)	Composted *Agaricus bisporus* (OC 25.9%, DOC 1.01%) Biomixture: SMS/straw/soil (25:50:25 *v*/*v*)	*K*_f_ adsorption values increased 1.3–7.7 times in the biomixture compared to soil. Desorption lower than 30%.	Karanasios et al. (2010) [41]
Thiabendazole Imazalil Ortho-phenylphenol Diphenylamine	Clay loam (OC 1.05%, pH 7.55)	*Pleurotus ostreatus* (OC 71%, pH 6.83) Biomixtures: SMS/soil (50:50 *v*/*v*) SMS/straw/soil (50:25:25 *v*/*v*) straw/SMS/soil (50:25:25 *v*/*v*)	Higher adsorption in the biomixtures than in the soil.	Karas et al. (2015) [42]

^1^ HA/FA, humic acids/fulvic acids. PI, polarity index. OC, organic carbon.

**Table 4:** Mobility of pesticides in SMS-amended soils.

Pesticide	Soil	SMS Type/Dose	Experimental Design	Results	Reference
Metalaxyl Penconazole	Sandy clay loam (OC 0.6%–1.47%, pH 7.7–7.8)	*Agaricus bisporus* fresh (F-SMS) and composted (C-SMS) F-SMS (OC 28.8%, DOC 3.83%) C-SMS (OC 27.4%, DOC 1.19%) Dose: 25 t·ha^−1^	Undisturbed soil cores: 40 cm (length) *x* 9 cm (i.d.) Pesticide dose: 10 mg–2.5 mg·kg^−^^1^ Leaching flow: 50 mL–0.8 cm water every day (unsaturated flow). Total volume: 1500 mL (2.5–4.5 PV)	Metalaxyl: Decreasing of leaching peaks up to 24-fold, and increased retention in columns in C-SMS > F-SMS. Penconazole: No leaching, 100% in columns (>60% in the upper layer in C-SMS).	Marín-Benito et al. (2009) [51]
Tebuconazole Azoxystrobin	Sandy loam (OC 1.31%,pH 7.7) Sandy loam (OC 1.37%,pH 7.7)	*Agaricus bisporus: Pleurotus* spp. (3:1) (OC 27.1%, DOC 1.22%, pH 7.1) Dose: 40 and 100 t·ha^−1^ *Agaricus bisporus: Pleurotus* spp. (3:1) (OC 27.1%, pH 7.1). Dose: 50 and 150 t·ha^−1^	Field experiments Tebucoazole dose: 0.25 and 1.25 kg·ha^−^^1^ Azoxystrobin dose: 0.25 and 1.25 kg·ha^−^^1^	Increased amounts of fungicides in the soil + SMS profile (0–50 cm) at different times. Amounts up to 20 cm (tebuconazole) and 50 cm (azoxystrobin) over 1 year.	Herrero-Hernández et al. (2011) [34] Herrero-Hernández et al. (2015) [33]
Linuron Diazinon Myclobutanil	Sandy loam (OC 0.47%, pH 7.9)	*Agaricus bisporus: Pleurotus* spp. (3:1) (OC 26.7%, DOC 1.98%, pH 7.1) Dose: 5% *w*/*w* (25 t·ha^−1^)	Packed soil columns: 3 cm (i.d.) × 20 cm (length) Pesticides dose: 1 mg Leaching flow: 500 mL/day of water (12–13 PV) (saturated flow) at a constant flow rate of 1 mL·min^−^^1^	Leaching peaks of pesticides in soil + SMS were smaller than in soil and at greater PV. SMS decreased leaching for myclobutanil > linuron > diazinon.	Marín-Benito et al. (2013) [52]
Tebuconazole Cymoxanil	Sandy clay loam (OC 0.67%, pH 7.52)	*Agaricus bisporus* (OC 24.5%, DOC 1.91%, pH 6.97) Dose: 5% and 50% *w*/*w*	Packed soil columns: 3 cm (i.d.) × 20 cm (length) Pesticides dose: 1 mg Leaching flow: 500 mL of CaCl_2_ (0.01 M) solution (12–13 PV), (saturated and saturated -non saturated (25 mL/day) flow)	Tebuconazole: Amounts leached decreased 2–3 times in soil + SMS 5 and soil + SMS 50 with both flows. Cymoxanil: Leached amounts only decreased in soil + SMS5 and soil + SMS50 when flow was saturated-non saturated (1.3–2.6 times).	Álvarez-Martín et al. (2014) [53,54]
Imazalil Ortho-phenylphenol	Clay loam (OC 1.05%, pH 7.55)	*Pleurotus ostreatus* (OC 20.6%, pH 6.83) SMS/straw/soil (50:25:25 *v*/*v*)	Packed soil columns: 12.5 cm (i.d.) × 90 cm (length)	Leaching of wastewater fungicides from citrus fruit-packaging plants decreased at <1% and <5%.	Karas et al. (2016) [55]

**Table 5:** Dissipation of pesticides in SMS-amended soils. OM, organic matter.

Pesticide	Soil	SMS Type/Dose	Results	Reference
Tebuconazole	Sandy loam (OC 1.31%, pH 7.7)	*Agaricus bisporus: Pleurotus* spp. (3:1) (OC 27.1%, pH 7.1). Dose: 40 and 100 t·ha^−1^	Fungicide dissipation was more rapid in amended soils than in unamended ones.	Herrero-Hernández et al. (2011) [34]
Azoxystrobin	Sandy loam (OC 1.37%, pH 7.7)	*Agaricus bisporus: Pleurotus* spp. (3:1) (OC 27.1%, pH 7.1). Dose: 50 and 150 t·ha^−1^	Lower fungicide dissipation was found in laboratory versus field experiments.	Herrero-Hernández et al. (2015) [33]
Iprovalicarb Metalaxyl Penconazole Pyrimethanil	Sandy clay loam (OC 0.59%, pH 7.87)	*Agaricus bisporus: Pleurotus sp.* (3:1) (OC 26.7%, pH 7.5). Dose: 10% *w*/*w* *Lentinula edodes* or Shiitake (OC 31.2%, pH 4.5) Dose: 10% *w*/*w*	Degradation rate was reduced for all fungicides in the soil amended with the composted SMS, and for iprovalicarb and penconazole in fresh SMS-amended soil.	Marín-Benito et al. (2012) [57]
Linuron Diazinon Myclobutanil.	Sandy loam (OC 0.47%, pH 7.9)	*Agaricus bisporus: Pleurotus sp.* (3:1) (OC 26.7%, pH 7.1) Dose: 5% *w*/*w* (25 t ha^-1^)	Dissipation increased (linuron) or decreased (diazinon and myclobutanil) in SMS-amended soil.	Marín-Benito et al. (2014) [58]
Tebuconazole Cymoxanil	Sandy clay loam (OC 0.67%, pH 7.52)	*Agaricus bisporus* (OC 24.5%, pH 6.97) Dose: 5% and 50% *w*/*w*	Fungicide dissipation rate was higher in the SMS-amended soil than in the unamended one.	Álvarez-Martín et al. (2016) [59]
Pirimicarb Azoxystrobin	Sandy loam (OC 0.89%, pH 7.49)	*Agaricus bisporus* (OC 24.5%, pH 6.97). Dose: 2% and 5% *w*/*w*	SMS facilitated the degradation of pirimicarb at both concentrations and of azoxystrobin at the lower concentration.	Álvarez-Martín et al. (2016) [60]
Thiabendazole, Imazalil Ortho-phenylphenol Diphenylamine Ethoxyquin	Clay loam (OC 1.05%, pH 7.55)	*Pleurotus ostreatus* (OC 71.0%, pH 6.83) Mixtures: SMS/soil (50:50 *v*/*v*) SMS/straw/soil (50:25:25 *v*/*v*/*v*) Straw/SMS/soil (50:25:25 *v*/*v*/*v*)	SMS rich organic biomixtures, such as SMS/straw/soil (50:25:25) and SMS/soil (50:50), showed the highest dissipation potential for all pesticides particularly of thiabendazole and imazalil.	Karas et al. (2015) [42]
Chlorothalonil Imidacloprid	Sandy loam (OC 1.96%, pH 7.84)	*Pleurotus eryngii* (OC 43.79%, pH 5.71) *Flammulina velutipes* (OC 44.44%, pH 7.76) *Lentinula edodes* (OC 39.40%, pH 5.09) Mixtures: Soil/straw/SMS (25:50:25 *v*/*v*/*v*)	Microbial activities and pesticide dissipation in SMS biomixtures were comparable to the original biobeds which include peat in their composition.	Gao et al. (2015) [61]
Metribuzin Buprofezin Azoxystrobin Iprodione, Dimethoate Indoxacarb Terbuthylazine	Sandy clay loam (OC 1.8%, pH 6.7)	*Pleurotus ostreatus* (OC 71.0%, pH6.83) Biomixtures: SMS/soil (50:50 *v*/*v*) SMS/soil (15:85 *v*/*v*) SMS/soil (5:95 *v*/*v*)	The SMS-biomixture was highly efficient in degrading the pesticide mixture with degradation rates being correlated with the proportion of SMS in the biomixture.	Karanasios et al. (2010) [16]
Dimethoate, Indoxacarb, Buprofezin, Terbuthylazine, Metribuzin, Metalaxyl-M, Iprodione, Azoxystrobin	Sandy clay loam (OC 1.8%, pH 6.57)	*Agaricus bisporus* (OC 25.9%, pH 6.74) Biomixture: SMS/Soil/straw (1:1:2 *v*/*v*/*v*)	SMS could be an alternative to peat in biobed or biomixtures in southern Europe where it is largely available at no cost. For most of the pesticides the degradation rate increased in the SMS-biomixture.	Karanasios et al. (2010) [41]
DDT Heptachlor Heptachlor epoxide	Organic rich soil (OC 3.27%, pH 5.6)	*Pleurotus ostreatus* SMS/Soil (1:1 *w*/*w*)	SMS was efficient to bioremediate soil contaminated by DDT, heptachlor and heptachlor epoxide.	Purnomo et al. (2014) [62] Purnomo et al. (2010) [63]
Tricyclazole	Soil	*Pleurotus ostreatus* Soil/SMS (5:1 *w*/*w*)	Degradation of tricyclazole was enhanced in soil/SMS mixture compared with soil.	Liu et al. (2008) [64]
Phenanthrene	Fine loam (subsoil) (OC 3.8%, pH 5.1)	*Agaricus bisporus* (pH 8)	The SMS could be used for the biodegradation of contaminated soil.	Reid et al. (2002) [65]
PAH (14 compounds)	Sandy loam (OC 1.91%, pH 8.13)	*Agaricus bisporus* (OM 63.9%, pH 6.28). Dose: 20%(*w*/*w*)	SMS was effective for PAH biodegradation in multi-polluted soil.	García-Delgado et al. (2015) [66]
PAH (13 compounds)	Clay loam (OC 0.7%, pH 8.20)	*Agaricus bisporus* (OC 32.4%, pH 6.7). Dose: 20%(*w*/*w*)	Sterile SMS application to historically polluted soil removed 3-ring PAH.	García-Delgado et al. (2015) [67]
Atrazine	Loamy sand (OC 0.15% pH 8.5)	Mushroom spent (OC 12.2%, pH 6.9). Dose: 9 t ha^-1^	The SMS accelerated the degradation of atrazine.	Kadian et al. (2008) [68]
Chlorpyrifos	Sandy loam (OC 0.50% pH 8.5)	Mushroom spent (OC 12.2%, pH 6.9). Dose: 1% (*w*/*w*)	The application of SMS to soil increased DHA and pesticide dissipation.	Kadian et al. (2012) [69]
1,3-Dichloropropene Chloropicrin	Sandy loam (OC 0.7%)	Two SMS Dose: 5% *w*/*w*	Both SMS amendments decreased the DT_50_ values of fumigants in soil.	Qin et al. (2009) [70]
